# Exploring rise of pregnancy in Bangladesh resulting from contraceptive failure

**DOI:** 10.1038/s41598-022-06332-2

**Published:** 2022-02-11

**Authors:** Md Nuruzzaman Khan, M Mofizul Islam

**Affiliations:** 1 Department of Population Science, Jatiya Kabi Kazi Nazrul Islam University, Trishal, Mymensingh, Bangladesh; 2 Department of Public Health, La Trobe University, Melbourne, Australia

**Keywords:** Medical research, Epidemiology

## Abstract

The objective of this study was to determine how changes in pre-pregnancy contraceptive methods used between 2011 and 2017/18 contributed to the changes in pregnancy resulting from contraceptive methods failure in Bangladesh. We used 2011 and 2017/18 Bangladesh Demographic and Health Survey data. Pre-pregnancy contraceptive methods failure was our outcome of interest, which was determined using women’s response about whether they became pregnant while using contraceptives before the most recent pregnancy. The year of the survey was the main explanatory variable. Descriptive statistics were used to describe the characteristics of the respondents. The difference in contraceptive methods failure across the socio-demographic characteristics was assessed by Chi-squared test. Multilevel poison regressions were used to determine the changes in the prevalence ratio of contraceptive methods failure across the survey years. Contraceptive methods failure rate increased between the surveys, from 22.8% in 2011 to 27.3% in 2017/18. Also, male condom use increased by 2.8%, while withdrawal/periodic abstinence and/or other methods decreased by 2.9%. The failure rates in these two categories of contraceptive methods increased substantially by 4.0% and 9.0%, respectively. Compared to the 2011 survey, the prevalence ratio (PR) of contraceptive methods failure was 20% (PR 1.2, 95% CI 1.1–1.3) high in the 2017/18 survey. This PR declined 13% (PR 1.1, 95% CI 1.04–1.2) once the model was adjusted for women’s and their partner’s characteristics along with the last contraceptives used. This study provides evidence of increasing rates of pregnancy due to contraceptive failure in Bangladesh. Given that this type of pregnancy is known to cause adverse pregnancy outcomes, including abortion, pregnancy complications, maternal and early child morbidity and mortality, policy and programs are needed to reduce its prevalence. Effective coordination between the contraception providers at the healthcare facilities and the households and a proactive role of family planning workers to make couples aware of the effective use of contraceptives are recommended.

## Introduction

An estimated 121 million unintended pregnancies occur each year globally, and the vast majority (92%) occur in low- and lower-middle-income countries (LMICs)^1^. Around 45% of total pregnancies in LMICs are attributable to unintended pregnancies, which are gradually increasing due to the rising number of women in the reproductive age (15–49 years)^1^. Unintended pregnancy has direct consequences on maternal morbidity and mortality and results in 61% of 121 million abortions worldwide each year^1,2^. This number is likely to be higher in LMICs. However, there is a lack of relevant data because, in many settings of LMICs, abortion is considered a criminal offense unless intended to save women’s lives. These abortions cause a maternal death every eight minutes in LMICs. Around five million women are hospitalized every year because of abortion-related complications^1,2^. Moreover, around 13% of unintended pregnancies end with miscarriages^3^, which are associated with increased risks of depression, anxiety, and mortality at younger ages^4,5^. Continued unintended pregnancies, which constitute 38% of all unintended pregnancies in LMICs, are further associated with haemorrhage, sepsis, and injury during pregnancy and labor; and a lower rate of breastfeeding and immunization following delivery^6^. The underlying causes of continued pregnancies are lower uptake of intrapartum care, birthing, postpartum care^7^, and a rising prevalence of depression^6,8^. A low rate of postpartum care and a high rate of depression affect the postpartum contraceptive uptake and consistent use, leading to a subsequent unintended pregnancy^9^.

While most unintended pregnancies occur because couples do not use contraception, around 30% of unintended pregnancies in LMICs occur due to contraceptive failure^10,11^. This includes both contraceptive-related failure (i.e., contraceptives did not work as expected) and user-related failure (i.e., stemming from incorrect or inconsistent use), particularly where the use of long-acting contraceptive is relatively low^12^. The reasons are often cultural and/or religious, such as preventing pregnancy through contraceptives is considered “*killing a human being*”. This perception is common in LMICs, particularly among the disadvantaged population.

Bangladesh is often characterized by numerous social and religious misconceptions that hinder access to family planning services and contraceptive use, particularly long-acting contraceptives^9^. However, the country has achieved remarkable progress over these areas since its independence in 1971, particularly due to its strong family planning-providing networks. A year after the independence, its Ministry of Health bifurcated the main implementing agency into two separate wings: Director General of Health Services (DGHS) and Director General of Family Planning (DGFP)^13^. This initiative aimed to enhance family planning education (e.g., family size and knowledge of contraceptive benefits and side-effects) and distribute contraception free of charge through home-visit every 14 days^14^. Moreover, since 1976, the country allowed the non-governmental organizations to provide contraception and that has now become a major contributor mainly in remote areas through their 105,000 community health workers^15^. These initiatives helped the country to achieve a significant increase in family planning services and contraception use. For instance, contraception use was increased from 8% in 1975 to 52% in 2011^16^, which contributed to significant reductions in maternal and early child mortality^17^. However, since 2011, the growth of contraceptive use has lost its pace; for instance, contraception use was increased only 2% between 2011 and 2014 and then declined again to 2011’s level (52%) in 2017/18^18^. This contributed to a reduction in maternal and early child mortality in the later segments of the Millennium Development Goals era of 2000–2015^18,19^. This sloth is caused by complacency and hence a relatively low focus on family planning activities^20,21^ and is likely to affect a rising prevalence of contractive failure and associated pregnancy and birth. However, relevant data is lacking on this. We aimed to investigate how changes in pre-pregnancy contraceptive methods used between 2011 and 2017/18 contributed to the changes in pregnancy resulting from contraceptive failure.

## Methods

### Data

We used two rounds of the Bangladesh Demography and Health Survey (BDHS) data conducted in 2011 and 2017/18. The National Institute of Population Research and Training, an independent research organization, conducted this survey. The survey interviewed all ever-married women of reproductive age (15–49 years) in the selected households. Households were selected based on a two-stage stratified random sampling approach. At the first stage, 600 and 675 enumeration areas (EAs, also known as clusters) were selected for 2011 and 2017/18 surveys, respectively, from a list of 296,718 EAs with probability proportional to the EA size. Bangladesh Bureau of Statistics created this list of EAs during 2011 as part of 2011’s National Population Census. At the second stage of sampling, a systematic sample of 30 households on average was selected from each EA. This produced a list of 38,160 households in total, 18,000 households in the 2011 round and 20,160 households in the 2017/18 round, from which 18,072 and 20,127 women were interviewed, respectively. Of these women, data of 12,241 individuals were analyzed in this study – 6,667 women from the 2011 round and 5,574 women from the 2017/18 round. These women were selected with the following criteria: (i) reported at least one live birth or pregnancy termination within 5 years of the survey dates (most recent pregnancy in case of more than one pregnancy), and (ii) reported using contraceptives at the time of recent pregnancy. Women who were not using contraceptives because of an unmet need for contraception or they wanted to be pregnant were excluded. The BDHS collected these data by using a calendar approach, in which the reproductive history (including birth, pregnancy, monthly contraception use, pregnancy termination) of each woman was collected for five years prior to the survey. If women reported using more than one contraception method in a given month, then the most frequently used method was identified as the main contraceptive. The details of this sampling procedure can be found elsewhere^16,18^.

### Outcome variables

Contraceptive method failure was our primary outcome variable. This variable was generated using women’s responses on whether they had stopped using contraception because they became pregnant (ended with either a live birth or termination) in their most recent pregnancy. Women who reported they were not using contraception because they wanted to be pregnant were excluded. Some previous studies used this approach in identifying contraceptive method failure^22,23^. Other outcome variables were type of contraceptives women used in their most recent pregnancies: categorized as pills (yes, no), IUD/injectable/female sterilization (yes, no), male condoms (yes, no), and withdrawal/periodic abstinence/other methods (yes, no). We generated these categorise considering contraceptive norms in Bangladesh, contraceptive method efficacy, duration of effectiveness, and the WHO guideline^24^. A similar classification was used in previous studies in Bangladesh^25^ and France^23^.

### Explanatory variables

Survey years were our main explanatory variable. Other variables included were respondents’ age, education, working status, parity, pregnancy termination history, husband/partner’s education, wealth quintile, place of residence, and administrative division. The BDHS used principal component analysis to create the wealth quintile variable based on durable goods such as radio/television, bicycle, house building materials in the households^16,18^. Previously there were seven administrative divisions in Bangladesh, and this increased to eight in 2015 when a new division named Mymensingh was created, splitting the Dhaka division. Therefore, for the 2017/18 survey, we merged Dhaka and Mymensingh divisions under the category “Dhaka division” to make it consistent with the 2011 survey.

### Statistical analysis

We summarized the characteristics of women and their partners using numbers and proportions and used the Chi-squared test for determining significant differences between the two survey years. Women’s reasons for contraceptive discontinuation were compared separately for 2011 and 2017/18 surveys. The multilevel mixed-effect Poisson regression with robust variance was used to examine the changes in prevalence ratio of contraceptives use from 2011 to 2017/18 rounds. We used this model firstly because the odds ratio estimated using logistic regression from a cross-sectional study may significantly overestimate the relative risk when the outcome is common (e.g., prevalence > 10%)^26,27^. Secondly, in the datasets, individuals were nested within households; and households were nested within EAs. This nested data structure generated multiple hierarchies and dependencies. Our multilevel mixed-effects Poisson regression model accounts for these multiple hierarchies and dependencies in data, and the problem of overestimation^28^. Three models were run separately. Model 1 was run using only the survey years. Model 2 was run using the survey years and adjusted for women and their partners’ characteristics. In Model 3, additional adjustment was made with the types of contraceptive. Results were reported as Prevalence Ratio (PR) and 95% Confidence Interval (95% CI). All statistical tests were two-sided and a p-value < 0.05 was considered statistically significant. Statistical software package Stata (version 15.2) was used for all analyses.

### Ethical consideration

We analysed secondary data extracted from the Demography and Health Survey (DHS) program in de-identified form with permission to analyse. The survey was approved by the National Research Ethics Committee of Bangladesh and ICF Macro International. No other ethical approval is required to analyse this survey data.

## Results

Table 1 shows the overall and year-wise distribution of the characteristics of participants. Around two-thirds of respondents were 20–29 years old, and around half had completed secondary education. In the combined dataset, around 28% of respondents had a history of pregnancy termination. Almost all (98.6%) women had one or more children, 24.7% had one, 37.2% had two, and 36.8% had three or more children. Around 71% of women identified rural areas as their place of residence. The distribution of the characteristics was largely similar in the two survey rounds.

**Table 1:** Demographic and pregnancy-related characteristics of the study participants**.**

	Overall (%) (n = 12,241)	2011 (%) (n = 6,667)	2017/18 (%) (n = 5,574)	*P*
**Age, years**				
15–19	10.25	10.41	10.12	< 0.01
20–24	32.92	33.33	32.59	
25–29	29.85	30.07	29.67	
30–34	17.85	16.36	19.10	
35–39	6.68	7.04	6.39	
≥ 40	2.44	2.80	2.14	
**Education**				
Illiterate/pre-primary	11.29	16.70	6.78	< 0.01
Primary	28.55	28.64	28.48	
Secondary	47.14	45.65	48.39	
Higher	13.01	9.01	16.35	
**Working status**				
Yes	27.68	10.23	42.26	< 0.01
No	72.32	89.77	57.74	
**Pregnancy termination history**				
Yes	27.97	30.17	26.13	< 0.01
No	72.03	69.83	73.87	
Husband’s education*				
Illiterate/pre-primary	19.20	25.38	13.98	< 0.01
Primary	31.57	28.58	34.10	
Secondary	30.94	29.22	32.39	
Higher	18.29	16.82	19.53	
**Maternal parity**				
0	1.41	1.36	1.45	< 0.01
1	24.67	22.62	26.38	
2	37.17	35.43	38.62	
≥ 3	36.76	40.59	33.55	
**Wealth quintile**				
Lowest	19.64	19.43	19.82	< 0.01
Second	18.73	17.93	19.40	
Middle	19.03	20.06	18.16	
Fourth	21.12	20.73	21.44	
Highest	21.49	21.85	21.18	
**Place of residence**				
Urban	28.69	27.03	30.08	0.03
Rural	71.31	72.97	69.92	
Administrative division**				
Barishal	6.77	6.79	6.76	< 0.01
Chattogram	17.57	17.87	17.31	
Dhaka	34.83	32.49	36.79	
Khulna	11.39	11.61	11.21	
Rajshahi	13.81	15.96	12.02	
Rangpur	11.14	11.38	10.94	
Sylhet	4.49	3.89	4.98	

*Husband’s education (n = 12,159, of which 82 responses were missing).

**Mymensingh division was created after 2016 by splitting the Dhaka division, therefore it was not available in the 2011 survey. To bring consistency in the 2017/18 survey, we merged the data of these two divisions.

**Figure 1:**
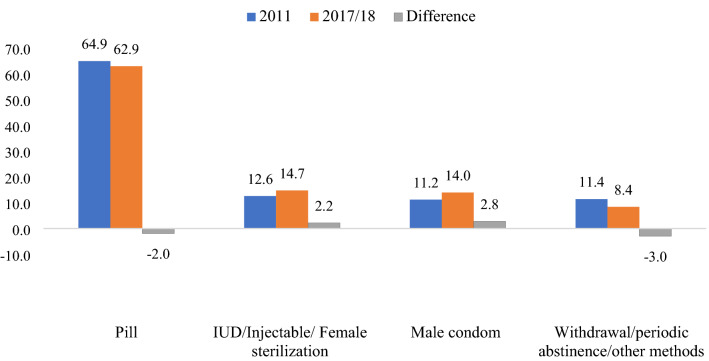
Pattern of contraceptives use during the last pre-pregnancy.

Figure 1 shows the types of contraception that women had been using at the month of becoming pregnant. Overall, pills were the dominant contraceptive and used by more than 60% of women. There were almost 3% (64.9% *vs* 62.9%), and 2% (11.4% *vs* 8.4%) declines in the prevalence of pills and withdrawal/periodic abstinence/other methods between 2011 to 2017/18 survey rounds, respectively. On the other hand, the use of male condoms and IUD/injectable/female sterilization had increased, at around 3% and 2%, respectively.

Reasons for stopping contraceptive use are summarized in Table 2. Around 46% of women in the 2011 survey and 41% of women in the 2017/18 survey reported that they had stopped using contraceptives because they wanted to have a child. Around 30% of women had stopped using contraceptives because of their husbands’ disapproval, concern over the side effects, lack of availability, cost, or infrequent sexual intercourse. Contraceptive failure was reported by 27% of women in the 2017/18 survey, a rise of 4% compared to the 2011 survey. Across contraceptive types, the failure rates were higher among women in the 2017/18 survey than the 2011 survey. The difference was the highest (almost 9%) among the women who had used withdrawal, periodic abstinence, or other methods (Table 2).

**Table 2:** Reasons for stopping the last pre-pregnancy contraceptives across methods type.

	2017/18					2011					*P* (2017/18 *vs* 2011)
	Number of women^1^ (%)	Desire to have a child (%)	Became pregnant (%)	Other reasons ^a^ (%)	*p*	Number of women^2^ (%)	Desire to have a child (%)	Became pregnant (%)	Other reasons ^a^ (%)	*p*	
**Total**	5574 (100)	2265 (40.63)	1521 (27.29)	1788 (32.08)	< 0.01	6662 (100)	3079 (46.22)	1521 (22.83)	2062 (30.95)	< 0.01	< 0.01
**Last contraceptive type**											
Pills	3615 (100)	1579 (43.68)	939 (25.97)	1097 (30.34)		4191 (100)	1985 (47.35)	973 (23.23)	1233 (29.42)		< 0.01
IUD/Injectable/ Female sterilization	701 (100)	268 (38.18)	118 (16.84)	315 (44.98)		982 (100)	503 (51.19)	114 (11.61)	365 (37.20)		< 0.01
Male condoms	624 (100)	218 (34.92)	183 (29.32)	223 (35.76)		929 (100)	379 (40.75)	235 (25.31)	315 (33.94)		< 0.01
Withdrawal/ periodic abstinence/ other methods	635 (100)	201 (31.58)	281 (44.32)	153 (24.11)		560 (100)	214 (38.12)	198 (35.38)	148 (26.50)		< 0.01

^1^1651 women did not use contraception in 2011 survey.

^2^1954 did not use contraception in 2017/18 survey.

^a^Other reasons include: husband’s disapproval, side effects, access/availability, wanted more effective methods, inconvenient to use, infrequent sex/husband away, cost, fatalistic, difficult to get pregnant.

***All row percentage are weighted.

Bivariate analysis of contraceptive failure is presented in Table 3. More than half of the women who reported contraceptives failure were in the 20–29-year age group in both survey rounds. The experience of pregnancy relating to contraceptive failure increased with maternal parity. Significant differences were found among women who had reported contraceptive failure across women’s and their partners’ educational status, history of pregnancy termination, and administrative division of their residence (Table 3).

**Table 3:** Distribution across demographic characteristics of women who experienced contraceptives failure.

	2017/18			2011		
	Total	%	*p*	Total	%	*p*
**Age, years**						
15–19	140	9.18	< 0.01	135	8.90	< 0.01
20–24	400	26.27		466	30.61	
25–29	468	30.79		389	25.58	
30–34	287	18.88		326	21.42	
35–39	160	10.56		151	9.93	
≥ 40	66	4.31		54	3.56	
**Education**						
Illiterate/pre-primary	317	20.83	< 0.01	141	9.27	
Primary	447	29.37		499	32.78	< 0.01
Secondary	653	42.95		659	43.34	
Higher	104	6.85		222	14.61	
**Working status**						
Yes	152	10.01	0.81	705	46.37	< 0.01
No	1369	89.99		816	53.63	
**Pregnancy termination history**						
Yes	620	40.76	< 0.01	575	37.80	< 0.01
No	901	59.24		946	62.20	
**Husband’s education**						
Illiterate/pre-primary	420	27.60	< 0.01	250	16.71	< 0.01
Primary	432	28.41		532	35.60	
Secondary	453	29.81		459	30.74	
Higher	216	14.19		253	16.95	
**Maternal parity**						
0	21	1.37	< 0.01	28	1.83	< 0.01
1	248	16.27		300	19.72	
2	473	31.09		530	34.85	
≥ 3	780	51.26		663	43.60	
**Wealth quintile**						
Lowest	299	19.63	0.43	317	20.84	0.39
Second	274	18.02		320	21.06	
Middle	302	19.89		271	17.85	
Fourth	330	21.70		316	20.73	
Highest	316	20.76		297	19.52	
**Place of residence**						
Urban	446	29.31	0.04	472	31.00	0.32
Rural	1075	70.69		1050	69.00	
**Administrative division**						
Barishal	85	5.60	0.01	95	6.21	0.05
Chattogram	265	17.38		247	16.24	
Dhaka	527	34.67		560	36.79	
Khulna	157	10.34		166	10.95	
Rajshahi	241	15.81		176	11.59	
Rangpur	182	11.97		185	12.16	
Sylhet	64	4.23		92	6.07	

In the combined dataset, the prevalence ratios of contraceptive failure among women who participated in the 2017/18 survey in reference to women in the 2011 survey are presented in Table 4. There was a 20% (95% CI, 1.11–1.29) increase in contraceptive failure in the 2017/18 survey compared to the 2011 survey. The prevalence ratio declined to 15% (95% CI, 1.06–1.25) once the demographic characteristics of women and their partners had been added to the model (Model A). The prevalence ratio declined further to 13% (95% CI, 1.04–1.23) in the final model (Model B) when types of contraceptives used at the time of becoming pregnant had been added. The prevalence of contraceptive failure was significantly higher among women who had used pills (PR, 1.98; 95% CI, 1.70–2.31), whose partners used male condoms (PR, 2.47; 95% CI, 2.06–2.96), or who used withdrawal/periodic abstinence/other methods (PR, 2.96; 95% CI, 2.51–3.50) than those who had used IUD/injectables/female sterilizations.

**Table 4:** Prevalence ratio for contraceptives failure across survey rounds and types of pre-pregnancy contraceptives use.

Variable	Contraceptive failure					
	Crude PR (95% CI)	*p*	Model A		Model B	
			aPR^1^ (95% CI)	*p*	aPR^2^ (95% CI)	*p*
**Year**						
2011 (Reference)	1.00		1.00		1.00	
2017/18	1.20 (1.11–1.29)	< 0.01	1.15 (1.06–1.25)	< 0.01	1.13 (1.04–1.23)	< 0.01
**Last contraception method**						
IUD/Injectable/Female sterilization (Ref)					1.00	
Pills					1.98 (1.70–2.31)	< 0.01
Male condoms					2.47 (2.06–2.96)	< 0.01
Withdrawal, periodic abstinence or other methods					2.96 (2.51–3.50)	< 0.01
**Age of the respondents**						
15–19 (ref)			1.00		1.00	
20–24			0.78 (0.67–0.90)	< 0.01	0.81 (0.70–0.94)	< 0.01
25–29			0.69 (0.58–0.81)	< 0.01	0.72 (0.61–0.86)	< 0.01
30–34			0.70 (0.58–0.84)	< 0.01	0.72 (0.59–0.87)	< 0.01
35–39			0.85 (0.70–1.03)	0.09	0.87 (0.71–1.06)	0.163
40–49			0.81 (0.63–1.06)	0.12	0.81 (0.63–1.05)	0.162
**Respondents’ education**						
No education (ref)			1.00		1.00	
Primary			0.91 (0.81–1.02)	0.11	0.93 (0.83–1.04)	0.19
Secondary			0.88 (0.77–1.00)	0.05	0.89 (0.78–1.01)	0.07
Higher			0.96 (0.79–1.17)	0.69	0.91 (0.75–1.11)	0.37
**Respondents’ working status**						
No (ref)			1.00		1.00	
Yes			1.05 (0.96–1.14)	0.33	1.08 (0.99–1.18)	0.08
**History of pregnancy termination**						
No (ref)			1.00		1.00	
Yes			1.62 (1.50–1.75)	< 0.01	1.57 (1.46–1.69)	< 0.01
**Parity**						
No children (ref)			1.00		1.00	
1 Child			0.98 (0.76–1.27)	0.89	0.98 (0.76–1.26)	0.86
2 Children			1.29 (0.99–1.67)	0.06	1.34 (1.03–1.76)	< 0.05
3 And more children			1.81 (1.38–2.39)	< 0.01	1.91 (1.45–2.52)	< 0.01
**Husbands’ education**						
No education (ref)			1.00		1.00	
Primary			1.01 (0.92–1.12)	0.84	1.01 (0.92–1.11)	0.82
Secondary			1.05 (0.94–1.18)	0.38	1.03 (0.92–1.14)	0.63
Higher			0.95 (0.81–1.12)	0.58	0.90 (0.77–1.06)	0.20
**Wealth quintile**						
Poorest (ref)			1.00		1.00	
Poorer			1.09 (0.98–1.22)	0.12	1.08 (0.97–1.20)	0.15
Middle			1.03 (0.91–1.16)	0.64	0.99 (0.88–1.12)	0.88
Richer			1.05 (0.92–1.19)	0.49	1.01 (0.89–1.15)	0.86
Richest			0.96 (0.83–1.12)	0.64	0.91 (0.78–1.06)	0.23
**Place of residence**						
Urban (ref)			1.00		1.00	
Rural			0.88 (0.81–0.95)	< 0.01	0.87 (0.80–0.95)	< 0.01
**Place of region**						
Barishal (ref)			1.00		1.00	
Chattogram			1.03 (0.90–1.19)	0.63	1.01 (0.89–1.15)	0.85
Dhaka			1.14 (0.99–1.31)	0.07	1.10 (0.96–1.25)	0.18
Khulna			1.07 (0.93–1.25)	0.35	1.02 (0.89–1.17)	0.78
Rajshahi			1.09 (0.94–1.27)	0.26	1.07 (0.92–1.23)	0.38
Rangpur			1.18 (1.03–1.36)	< 0.05	1.17 (1.03–1.34)	< 0.05
Sylhet			1.19 (1.00–1.40)	< 0.05	1.07 (0.91–1.24)	0.43

*PR* Prevalence ratio, *aPR* Adjusted prevalence ratio, *CI* Confidence interval.

^1^Adjusted for maternal age, education, working status, pregnancy termination history, husband’s education, parity, wealth quintile, place of residence and region of residence.

^2^Adjusted for Model A covariates and last contraceptives method used prior to becoming pregnant.

We found prevalence ratio of contraceptive failure declined with the increase of women’s age from 15–19; however, they were only statistically significant for ages 20–24 (PR, 0.81, 95% CI, 0.70–0.94), 25–29 (PR, 0.72, 95% CI, 0.61–0.86), and 30–34 (PR, 0.72, 95% CI, 0.59–0.87). Women having a history of pregnancy termination reported a 57% (PR, 1.57, 95% CI, 1.46–1.69) increase in contraceptive failure compared to their counterparts who did not experience pregnancy termination. The prevalence ratio of contraceptive failure was also found to be higher among women having two children (PR, 1.34, 95% CI, 1.03–1.76) or three or more children (PR, 1.91, 95% CI, 1.45–2.52) compared to the women having no children. Around 13% declined prevalence ratio of contraceptive failure was reported for the rural women as compared to the urban women. Moreover, when we compared regions of residence, women residing in the Rangpur division reported around 17% (95% CI, 1.03–1.34) higher prevalence ratio of contraceptive failure compared to the women residing in the Barishal division.

## Discussion

This study provides new insight on how changes in pre-pregnancy contraceptive use affect the percentage of pregnancies resulting from contraceptive failure. We found a 20% rise in pregnancy attributed to contraceptive failure between the survey rounds. This indicates a pathway to increase the prevalence of unintended pregnancies and associated adverse outcomes, including maternal and child mortality, in Bangladesh, which will create challenges in achieving the Sustainable Development Goals (SDGs). In Bangladesh, there has been a change in perception over the last decade, and a growing number of families are now reluctant to take more than two children^18^. With rising levels of education and socio-economic development, couples are now more aware of their reproductive goals, comfortable about sharing their experiences of contraceptives use^29^, and reporting contraceptive failure^9^. This improved situation is congenial to implement programs to reduce unintended pregnancies attributed to contraceptive failure.

This study found a decline in the rates of traditional contraceptives such as pills, withdrawal, periodic abstinence. The failure rates for these contraceptives are usually relatively high, around 10% for pills, and 13% for withdrawal and periodic abstinence^30^. Although this decline in traditional contraceptives use suggests some progress Bangladesh has made over the years in terms of methods choices, this change, however, is insignificant compared to that in other LMICs^31–33^. Although modern contraceptive use, such as male condoms, IUD, injectable and female sterilization has increased over the years, the growth was insignificant. The failure rates of these modern contraceptives could be as high as they are for traditional methods if not used properly^30^. This may partly explain the small changes in the occurrence of pregnancy from contraceptive failure since 2011^16,18^. Although this small change in contraceptives uses pattern and their types are not unexpected, the noticeable change in contraceptive failure over the years is.

The programs for family planning and contraception in Bangladesh are guided by the five-year plan that was developed twice during this study period, the sixth five-year plan (2011–2015) and the seventh five-year plan (2016–2020)^20,21^. However, compared to the previous five-year plans, these two had a weaker focus on family planning and contraception promotion, and a stronger focus on intrapartum, birthing, and postpartum care aimed at reducing maternal and child mortality^20,21^. Such focuses were made to achieve, initially, the Millennium Development Goals (MDGs) and later the SDGs. During this period, there had been strategies to expand family planning services from healthcare centers. While the provision of family planning services at the household level remains functional, there appears to be less enthusiasm than it was during the 1990s and the first decade of the 2000s^9,34^. Also, a shortage of workers is common in both levels along with inadequate skills, monitoring, and supervision^34^, and these problems are becoming acute day by day. Consequently, there is a lower number of family planning visits at the household level than what is recommended (one visit in every 14 days), and poor-quality services about the effective use of contraceptives^35,36^.

Another concern is the drawbacks of the integrated approach of contraception services provided at the healthcare centers with other forms of maternal healthcare services^9,37,38^. In Bangladesh, contraception is considered culturally sensitive and warrants privacy for providers to discuss, albeit rarely facilities have such private corners^9^. Moreover, such provision of joint services makes healthcare centers overcrowded. Consequently, healthcare workers, for whom providing maternal healthcare is the priority, do not get enough time to discuss contraception, and counselling services^9,39^. This relatively low priority may result in women’s preferences for traditional, less effective but handy contraceptives than long-acting methods. Together, these indicate system-level challenges to contraceptive uptake and their consistent use, and thereby, an increased likelihood of contraceptive failure. Also, there exists little or no coordination between services being provided at household levels and healthcare centers. Such uncoordinated delivery results in unequal coverage, and some people may miss out on services^9,39^. This disjoint approach may also increase the risk of the contraceptive method mix, which is an important predictor of contraceptive failure^22,29^.

We found lower likelihoods of contraceptive failure among older women, although these were only significant among women up to the age group 30–34 years. This overall negative relationship could be explained by decreasing biological fecundity, or the probability of conception per coital act, with increasing age^40^. The coital frequency also decreases with age. Moreover, older women are more knowledgeable about effective contraceptives and their reproductive goals^41^. Further research is needed to examine the reasons for insignificant relationships for 35–49-year age groups. We also found a higher likelihood of contraceptive failure among women with higher parity. This association could be explained by the fact that women having a higher parity may not want any more children and thus some of the pregnancies at this stage were reported as unwanted and resulted from contraception failure. Finally, the observed association between a history of pregnancy termination and contraceptive failure was likely due to the termination of some of those pregnancies resulting from contraceptive failure.

The current study has several strengths. Firstly, we analyzed large nationally representative datasets collected in two separate time points. Secondly, the analytical approach we used considered the hierarchical structure of the data and avoided the chance of effect-size overestimation that may occur if conventional logistic regression is employed in cross-sectional studies. Thirdly, we adjusted the regression models for a wide range of confounding factors. This study also has some limitations. The analysis of cross-sectional data means we cannot determine the causal relationship between the exposure and outcome variables. Moreover, the data was collected based on women’s self-response on contraceptive use during five years prior to the survey dates. Remembering information of such a long duration may have caused recall bias and misreporting about the type of contraceptives women had used before their last pregnancies, although referring to pregnancy time, which is usually a memorable event, may have reduced this bias to some extent. In addition, we identified contraceptive methods failure in the most recent pregnancy by using women's responses on whether they had stopped using contraception because they became pregnant. Contraceptive use discontinuation in other previous pregnancies was not examined to count the contraceptive failure. Also, only one episode of pregnancy (the most recent one) was considered. Therefore, the actual estimate for all women will be considerably higher. However, restricting analyses only in the most recent pregnancy data helped us to reduce the recall bias.

## Conclusion

Traditional contraceptive methods use slightly declined in Bangladesh with a marginal increase in modern contraceptive methods. However, increased use in modern contraceptives in 2017/18 compared to the 2011 survey did not bring down but increased pregnancies from contraceptive failures among women. Policies and programs are needed to increase the availability of family planning services at the household level and to improve the coordination between the services provided at the healthcare centers and household levels.

## Data Availability

The datasets used and analyzed in this study are available from the Measure DHS website: https://dhsprogram.com/data/available-datasets.cfm.
